# Behavioral determinants of arsenic-safe water use among Great Plains Indian Nation private well users: results from the Community-Led Strong Heart Water Study Arsenic Mitigation Program

**DOI:** 10.1186/s12940-023-00965-0

**Published:** 2023-05-15

**Authors:** Kelly Endres, Tracy Zacher, Francine Richards, Lisa Bear Robe, Martha Powers, Joseph Yracheta, David Harvey, Lyle G. Best, Reno Red Cloud, Annabelle Black Bear, Steve Ristau, Dean Aurand, Leslie Skinner, Jamie Perin, Christa Cuny, Marie Gross, Elizabeth D. Thomas, Ana Rule, Kellogg Schwab, Lawrence H. Moulton, Marcia O’Leary, Ana Navas-Acien, Christine Marie George

**Affiliations:** 1 Department of International Health, Johns Hopkins Bloomberg School of Public Health, Baltimore, MD USA; 2 Missouri Breaks Industries Research, Inc., Eagle Butte, SD USA; 3 Indian Health Service, Rockville, MD USA; 4 Environmental Resource Department, OST, Pine Ridge, USA; 5 Mid Continent Testing Labs, Inc., Rapid City, SD USA; 6 Department of Environmental Health and Engineering, Johns Hopkins Bloomberg School of Public Health, Baltimore, MD USA; 7 Department of Environmental Health Science, Mailman School of Public Health, Columbia University, New York, NY USA

**Keywords:** Arsenic, Health behavior, Water treatment, Water, Private well

## Abstract

**Background:**

The objective of this study was to evaluate the behavioral determinants associated with exclusive use of arsenic-safe water in the community-led Strong Heart Water Study (SHWS) arsenic mitigation program.

**Methods:**

The SHWS is a randomized controlled trial of a community-led arsenic mitigation program designed to reduce arsenic exposure among private well users in American Indian Great Plains communities. All households received point-of-use (POU) arsenic filters installed at baseline and were followed for 2 years. Behavioral determinants selected were those targeted during the development of the SHWS program, and were assessed at baseline and follow-up.

**Results:**

Among participants, exclusive use of arsenic-safe water for drinking and cooking at follow-up was associated with higher self-efficacy for accessing local resources to learn about arsenic (OR: 5.19, 95% CI: 1.48–18.21) and higher self-efficacy to resolve challenges related to arsenic in water using local resources (OR: 3.11, 95% CI: 1.11–8.71). Higher commitment to use the POU arsenic filter faucet at baseline was also a significant predictor of exclusive arsenic-safe water use for drinking (OR: 32.57, 95% CI: 1.42–746.70) and cooking (OR: 15.90, 95% CI: 1.33–189.52) at follow-up. From baseline to follow-up, the SHWS program significantly increased perceived vulnerability to arsenic exposure, self-efficacy, descriptive norms, and injunctive norms. Changing one’s arsenic filter cartridge after installation was associated with higher self-efficacy to obtain arsenic-safe water for drinking (OR: 6.22, 95% CI: 1.33–29.07) and cooking (OR: 10.65, 95% CI: 2.48–45.68) and higher perceived vulnerability of personal health effects (OR: 7.79, 95% CI: 1.17–51.98) from drinking arsenic-unsafe water.

**Conclusions:**

The community-led SHWS program conducted a theory-driven approach for intervention development and evaluation that allowed for behavioral determinants to be identified that were associated with the use of arsenic safe water and changing one’s arsenic filter cartridge. These results demonstrate that theory-driven, context-specific formative research can influence behavior change interventions to reduce water arsenic exposure. The SHWS can serve as a model for the design of theory-driven intervention approaches that engage communities to reduce arsenic exposure.

**Trial registration:**

The SHWS is registered with ClinicalTrials.gov (Identifier: NCT03725592).

**Supplementary Information:**

The online version contains supplementary material available at 10.1186/s12940-023-00965-0.

## Background

Arsenic contamination in potable water has long been recognized as a serious public health concern globally [1]. Health impacts of prolonged elevated exposure to arsenic include skin, lung, and bladder cancers, cardiovascular disease, diabetes, and kidney disease, as well as developmental and cognitive impacts if exposed in utero or during early childhood [2–4]. These health effects are seen even at low to moderate levels of arsenic exposure [5]. The United States Environmental Protection Agency (EPA) defines the maximum contaminant level (MCL) for arsenic in potable water as 10 μg/L [6]. However, the EPA mandate only applies to public water sources; when private wells exceed the MCL of 10 μg/L, the burden of water treatment is left to private well users [7]. In the United States, a high income country, it is estimated that over 2.1 million individuals (5% of the population using private wells), remain exposed to drinking water arsenic levels above the MCL [7]. Rural communities, which generally have limited access to public water supplies, represent the majority of this population [8, 9]. American Indian communities, especially those in the Great Plains, Southwest, and Northeast, where groundwater arsenic contamination is common [10], are also disproportionately impacted due to their reliance on private water wells.

Reducing arsenic exposure from private wells is challenging. First, homeowners must be aware of their water quality. Arsenic is both tasteless and odorless, meaning that private well users will know their arsenic contamination levels only through water arsenic testing. Many studies have assessed factors influencing well water arsenic testing, identifying barriers such as a lack of awareness, lack of access to testing services, high prices for arsenic tests, and low perceived vulnerability [11–14]. Studies show that households are motivated to test for environmental contaminants in their water when there are perceived health risks or perceived changes in the taste, color, or smell of water, and when encouraged by the behaviors of others or to comply with social norms [11, 12, 15].

Significant barriers to reducing arsenic exposure in water used for drinking and cooking often stem from gaining access to, and the sustained use of, arsenic-safe water sources [16, 17]. Possible risk reduction methods include switching to an arsenic-safe public water source, installation of a point-of-entry (POE) or point-of-use (POU) water treatment system, or use of bottled water. However, many of these options require active participation in the installation, maintenance, and sustained use of the arsenic treatment option [18]. Furthermore, bottled water, typically an arsenic-safe option, can be associated with a significant financial burden for some households [19, 20]. Bottled water use also creates substantial plastics pollution and represents a high energy demand, limiting its viability as a long-term solution for the provision of clean water [21].

Only a handful of studies have assessed behavioral and situational factors associated with using and maintaining an arsenic removal device or other mitigation option to reduce arsenic exposure [17, 22–27]. Several studies have identified that knowledge of arsenic contamination alone is not sufficient to induce water treatment or use of an alternative arsenic-safe water source [22–24]. In one study conducted in rural Maine, investigators found that after households received water test results indicating arsenic concentrations > 10 μg/L, 45% of households installed arsenic treatment systems, 30% undertook an alternative mitigation option such as drinking bottled water, and 27% of households took no protective measures [25]. Perceived risk and well water arsenic concentration were significant motivations for taking protective action. Common reasons for not taking protective action were lack of concern and high cost of arsenic mitigation options, with perceived cost influenced by an individual’s perceived risk of drinking arsenic contaminated water. Another study conducted in New Jersey reported similar findings, with 54% of high arsenic households using water treatment, 10% exclusively using bottled water, and 37% of households taking no arsenic mitigation measures [26]. Those who took protective actions to reduce arsenic exposure had higher levels of perceived risk of arsenic exposure. Perceived susceptibility, perceived barriers, self-efficacy, and commitment all significantly predicted use of arsenic mitigation measures. Outside of the United States, several studies have evaluated the use of arsenic-safe water sources using randomized controlled trials (RCTs) [28, 29]. However, no RCT, to the authors’ knowledge, has been conducted in the United States to evaluate an arsenic mitigation program.

In this study, we assessed the behavioral determinants associated with arsenic-safe water use based on the Risks, Attitudes, Norms, Abilities, and Self-regulation (RANAS) model of health behavior change [30]. This model was developed to target psychosocial factors influencing water, sanitation, and hygiene (WASH) behaviors. Behavioral determinants are categorized into five main factors: risk, attitude, norm, ability, and self-regulation. Each factor focuses on intentions, use, and habits that may influence WASH behaviors. Factors are evaluated in social, physical, and personal contexts and each factor is associated with a unique behavior change technique. Previous studies have used the RANAS model to assess arsenic-safe water use behavior, and supported the development of theory-informed approaches for interventions focused on reducing arsenic exposure in Bangladesh [25, 26, 28, 29, 31].

The Strong Heart Water Study (SHWS) is an RCT of a multi-level, community-led arsenic mitigation program to reduce arsenic exposure in drinking and cooking water among the Lakota and Dakota Nations in the American Great Plains region [32]. The SHWS is an extension of the Strong Heart Study, a decades long program investigating cardiovascular disease and other health topics in partnership with American Indian communities. Previous work from the Strong Heart Study has found associations between water arsenic exposure and health impacts including cancers, cardiovascular disease, and diabetes prevalence and control [33–42]. Water arsenic exposure is occurring in the context of historical environmental injustices faced by American Indian communities [20]. Initial water quality assessments for the SHWS indicated that over 25% of private well users in our partner communities are exposed to arsenic concentrations ≥10 μg/L, highlighting the importance of effective interventions for these communities [43]. Formative research for the SHWS found that awareness and concern about water arsenic contamination was present but varied among participants [44]. Community members noted safety, cost, and water quality factors such as taste and color were important considerations for selecting water for drinking and cooking.

The primary aim of this study was to prospectively evaluate the behavioral determinants associated with exclusive arsenic-safe water use in the SHWS. The secondary aims were to measure changes in behavioral factors from the beginning to end of the intervention and determine the impact of behavioral determinants on arsenic filter change. This is the first RCT of a water arsenic intervention program in the Americas.

## Methods

### Study design

The SHWS is a collaboration between three Great Plains Nations, the Indian Health Service (IHS), Columbia University, and the Johns Hopkins Bloomberg School of Public Health. This study utilized a two-arm cluster RCT design to determine the effectiveness of a multi-level community-led arsenic mitigation program in reducing arsenic exposure from water used for drinking and cooking (ClinicalTrials.gov Identifier: NCT03725592). Household enrollment took place between July 2018 and November 2019. Final follow-up visits took place between November 2020 and November 2021.

### Eligibility criteria

To be eligible for participation in the SHWS, households had to be located in a Great Plains Nation, have at least one American Indian household member residing in the home, and utilize a private well for drinking and cooking with an arsenic concentration > 10 μg/L and uranium concentrations < 30 μg/L. Uranium contamination below the EPA MCL for uranium was included because the arsenic filters provided by the SHWS do not remove uranium (30 μg/L) [32, 43]. Households with high uranium were provided with resources to seek alternative water treatment options. An extensive overview of the initial water quality and eligibility assessment for the SHWS has been reported previously [43]. Multiple participants could be enrolled per household. After household enrollment, eligible household members (≥ 12 years of age with the household as their primary residence) were enrolled.

### POU arsenic filter installation and water sampling

Missouri Breaks Industries Research, Inc. (MBIRI), a local American Indian owned and led research organization, managed and organized study activities. After enrollment, each study household received a Multipure® (Model CB-As-SB, Las Vegas, NV) POU arsenic filtration system installed under the kitchen sink. Filter installation was completed by community members working at the Tribal Housing Authority in partnership with the IHS. At installation, households were provided with device use instructions and one replacement filter cartridge. A cartridge change was recommended every 12 months. The POU arsenic filter was connected to a filter faucet installed alongside the kitchen faucet for drinking and cooking water use. Based on feedback from the pilot study, the POU arsenic filter faucet was also connected to the refrigerator water and icemaker on request [32]. Other activities such as washing dishes, cleaning, and washing hands could still be conducted using the kitchen faucet to reduce the burden on the POU arsenic filter device and lengthen the life of each cartridge. Kitchen faucet samples were collected at baseline and 1-month, 6-month, and long-term follow-up visits (1 to 2 years after installation). POU arsenic filter faucet water samples were collected at filter installation and each follow-up visit. Water samples were analyzed at the Mid-Continent Testing Labs, Inc. (Rapid City, South Dakota) by inductively coupled plasma mass spectrometry (ICP-MS). A comprehensive description of water sample collection and analysis has been published elsewhere [43].

### Questionnaire

At baseline and follow-up visits, each participant was administered an in-person structured questionnaire on demographics, behavioral determinants, water use, and other study factors. Questionnaire interviews were conducted by trained research assistants from MBIRI. Due to the COVID pandemic, some visits were delayed, with an average duration between baseline and final follow-up of 2 years. The primary participant in each household was additionally administered a household-level questionnaire assessing the condition of the POU arsenic filter, technical issues related to use, and if the arsenic filter cartridge was changed since the previous visit.

### Behavioral determinants

The community-led SHWS program was designed to target the behavioral determinants of arsenic-safe water use in the RANAS model [44]. Behavioral determinants were assessed on an individual level using Likert scale items based on the psychosocial factors in this model. The following behavioral determinants were assessed: perceived vulnerability, descriptive norms, injunctive norms, self-efficacy, commitment strength, instrumental attitudes, and knowledge. In addition, other behavioral determinants not explicitly defined in the RANAS model were included based on previous studies and our qualitative research findings: perceived cost, competing priorities, perceived safety of tribal water system, perceived extent of contamination in the community, and user preferences [32, 45]. Perceived cost, perceived vulnerability, competing priorities, arsenic knowledge, and self-efficacy regarding aspects of arsenic filter cartridge cost and replacement were all behavioral determinants included based on pilot study qualitative findings [44]. Behavioral determinant questionnaire items are described in Table 1. All items except those assessing arsenic knowledge were coded using a 1–5 Likert scale (e.g. 1 = “strongly disagree” or “0% sure” to 5 = “strongly agree” or “100% sure”). Additional details on response options are included in Supplementary Table 1. Four knowledge items were included in the questionnaire with an open-ended response format. The items were: “Name two health conditions that can happen from arsenic exposure” (1 point for each correct response, total possible score of 2 points), “Name two tasks where it is OK to use water with high arsenic” (1 point for each correct response, total possible score of 2 points), “Name two tasks where it is NOT OK to use water with high arsenic” (1 point for each correct response, total possible score of 2 points), and “How could you remove arsenic from drinking water” (1 point for a correct response, a total possible score of 1 point). An overall arsenic knowledge variable was calculated by adding scores from the 4 knowledge items for a total of 7 points. All behavioral determinants were rescaled from original coding to a 0–1 scale to standardize answers and improve interpretability.

**Table 1:** Example behavioral determinants measured in the SHWS, and corresponding behavior change techniques delivered in the SHWS intervention

Type of factor	Factor	Definition	Example behavior change technique delivered	Example items	Hypothesized change
Risk factors	Perceived vulnerability	Perceived risk of health problems from arsenic exposure [46]	All: Phone calls at 1-week, and 1-, 3-, and 5-months after arsenic filter installation to provide information on the health risks associated with arsenic exposure. Intensive: Video testimonials about the health risks associated with exposure to arsenic through drinking water, including cancers, heart disease, and diabetes.	How high or low are the chances of you getting health problems from arsenic if it is in your well water?	Higher perceived vulnerability
				Because no one in my house has developed health problems from arsenic, I am not concerned about arsenic in my well water.	Higher perceived vulnerability (lower score = greater concern)
	Arsenic knowledge	Understanding of the properties of arsenic, and comprehension of the health effects from water arsenic exposure and related mitigation options	All: FAQ and letter to the household including information about arsenic, health effects of arsenic exposure, and mitigation options. Intensive: Video testimonials about the properties of arsenic (e.g., cannot be seen, smelled, or tasted), health risks associated with exposure to arsenic through drinking water, behaviors that may increase exposure to arsenic (e.g., boiling arsenic unsafe water for consumption), and mitigation options (e.g., testing private well water for arsenic, installing an arsenic filter, and drinking and cooking with arsenic safe water).	Name two health conditions that can happen from arsenic exposure.	Higher arsenic knowledge
				Name two tasks where it is NOT OK to use water with high arsenic.	Higher arsenic knowledge
				How could you remove arsenic from drinking water?	Higher arsenic knowledge
Norm factors	Descriptive norms	Perceptions about the behaviors commonly performed by others [47]	All: Informing households that the same intervention was provided to other households with elevated arsenic in their community to improve water quality for people with private wells. Intensive: Video testimonials about community members testing private wells for arsenic, installing an arsenic filter, and using arsenic safe water for drinking and cooking.	How many people in your community with arsenic in their wells drink this water without filtration?	Lower descriptive norms
				How many people in your community with arsenic in their wells use an arsenic filter for their drinking water?	Higher descriptive norms
	Injunctive norms	Perceptions about behaviors that others commonly approve or disapprove of [48]	Intensive: Video testimonials from community elders encouraging testing private wells for arsenic, installing an arsenic filter, and using arsenic safe water for drinking and cooking.	How much would people who are important to you approve or disapprove of you using water containing high arsenic for drinking?	Higher injunctive norms (increased disapproval = lower score)
Ability factors	Self-efficacy	The belief in one’s abilities to confront and manage possible situations [49]	All: Phone calls at 1-week, and 1-, 3-, and 5-months after arsenic filter installation to troubleshoot challenges households faced when using the arsenic filter, discuss strategies to overcome challenges, and provide suggestions for how to facilitate using water from the filter faucet for all drinking and cooking needs. Intensive: Video testimonials from community members about trouble-shooting arsenic filter use; Important Reminders Sheet with advice on how to make sure one is able to use arsenic safe water for drinking and cooking.	How sure are you that you can use your arsenic filter faucet every time you need water for drinking in your home?	Higher self-efficacy
			All: Provision of one replacement arsenic filter cartridge and written instructions (device manual) on how to change the filter cartridge; phone calls at 3-, and 5-months after arsenic filter installation with reminders of when to change the filter cartridge and to refer to provided instructions. Intensive: Step-by-step video on how to change the arsenic filter cartridge, as demonstrated by a community member; making an action plan for when to change the arsenic filter cartridge.	How sure are you that you yourself can change your arsenic filter cartridge when needed?	Higher self-efficacy
	Commitment strength	Level of dedication to the decision to perform a behavior [50]	Intensive: Video testimonial from an arsenic filter user about using the filter despite challenges (e.g., slow water flow).	How committed do you feel to drinking water only from your arsenic filter faucet?	Higher commitment strength
Other	Perceived cost	The belief about the true cost of a behavior and its alternatives [45]	All: Phone calls at 1-week, and 1-, 3-, and 5-months after arsenic filter installation to explain how to maintain the arsenic filter over time.	I can afford to fix my arsenic filter if it breaks.	Decreased perceived costs (higher score = decreased perceived cost)
	Competing priorities	Priority of adopting a new behavior (or behavior change) compared to competing concerns or goals (that may require the same resources) [51]	Intensive: Important Reminders sheet with advice on time-saving techniques to manage time to use the arsenic filter faucet when there might be competing priorities in the home.	Of all the things I have to worry about, the arsenic filter is not at the top of my list.	Increased prioritization of the arsenic filter (lower score = greater prioritization)
	Perceived safety of tribal water system	The belief that the municipal water system provides safe water [45]	All: FAQ stating that the tribal municipal water supply is monitored to make sure it meets EPA standards for arsenic. Intensive: Video testimonial from community elder stating that the municipal water is arsenic safe.	The tribal water system in my area has a safe level of arsenic.	Increased perceived safety
	Perceived extent of contamination in community	The belief that arsenic is present in water sources in the community [45]	All: FAQ stating that arsenic above the EPA standard has been found in private wells in the partner community; providing households with contact information for the wellcare® Hotline for additional information on arsenic contamination in private wells.	How many people in your community have arsenic in their wells?	Increased perceived extent contamination
	User preferences	Preference for one safe water source over another [45]	Intensive: Provision of travel water bottles to fill with arsenic safe water from the filter faucet for convenience.	If given the choice, I would prefer bottled water over water from an arsenic filter.	Decreased preference for bottled water

### Water use

Water arsenic exposure was assessed at the individual level as the self-reported use of arsenic-safe drinking and cooking water sources in the past month at baseline and follow-up visits. Arsenic-safe water sources included the use of the POU arsenic filter faucet, bottled water, or the municipal water system, and arsenic-unsafe sources included use of the kitchen faucet, bathroom faucet, and refrigerator filter or icemaker (if not reported to be connected to the POU arsenic filter faucet). Participants were also asked about the type of water use in the past month for drink and food items. Drink items included homemade tea or coffee; homemade juices (e.g., fruit punch, lemonade, Kool-Aid); powdered milk; and homemade ice. Food items included homemade soup or stew; bread, muffins, pancakes, cake, cookies, or waffles; pasta, grains, or boiled vegetables; rice made with water; and gravy made with water. For analysis, water use was assessed using the following variables: 1) exclusive use of arsenic-safe water for drinking, 2) exclusive use of arsenic-safe water for cooking, and 3) exclusive use of arsenic-safe water for drinking and cooking.

### Intervention

The community-led SHWS program includes a community water arsenic testing program, two distinct household-level health communication programs (SHWS mobile health (mHealth) & filter arm vs. SHWS intensive arm), and provision of a POU arsenic filter faucet (for all households). The SHWS mHealth & filter arm program provides households with a POU arsenic filter faucet and 3 calls to promote filter use and maintenance at 2 weeks and 3 and 5 months after filter installation by a community promoter. The SHWS intensive program provides the same filter, as well as 3 phone calls and 3 Facebook messages at 2 weeks and 3 and 5 months after filter installation, and 3 in-person home visits at 1 week, 1 month, and 6 months after filter installation by a community promoter. Comprehensive descriptions of the study intervention and COVID-19 related changes have been published elsewhere (George et al. submitted).

### Statistical analysis

Follow-up analyses were based on the final household visit for each participant. If a participant had both a 6-month and long-term follow-up visit, the long-term follow-up visit was used. To compare changes in behavioral determinants from baseline to follow-up, descriptive statistics were calculated. Logistic regression with generalized estimating equations (GEE) with exchangeable working correlation was performed to account for clustering within households. Study timepoint was the outcome, with each behavioral determinant as the predictor. Logistic regression models with GEE with exchangeable working correlation were also used to examine the baseline behavioral determinants associated with the use of arsenic-safe water for drinking and cooking with household as the cluster, baseline behavioral determinant or demographic factor as the predictor, and follow-up water use as the outcome. To assess the influence of baseline behavioral determinants on arsenic filter cartridge change during the study period, GEE logistic regression models with independent working correlation (due to the smaller sample size) were run with behavioral determinants at baseline as the predictors and arsenic filter cartridge change as the outcome. Finally, logistic regression models with GEE with exchangeable working correlation were also used to assess the influence of the change in baseline determinants (follow-up – baseline) over the study period on use of arsenic-safe water with household as the cluster variable. All analyses were completed using SAS software (version 9.4, Cary, NC).

## Results

A total of 84 participants were enrolled at baseline from 50 households, of whom 75 completed a long-term or 6-month follow-up visit (11% loss to follow-up). At baseline, 51 participants from 27 households were enrolled in the SHWS mHealth & filter arm and 33 participants from 23 households in the SHWS intensive arm. Forty-seven participants in the SHWS mHealth & filter arm and 28 participants in the SHWS intensive arm completed a 6-month or long-term follow-up visit for loss to follow-up of 8% and 15%, respectively. Higher intensive arm enrollment later in the enrollment period resulted in greater COVID-19 disruption accounting more higher loss to follow-up in that arm. The mean age at baseline was 54 years (± standard deviation (SD), min-max: 19, 13–85) and 54% of participants were female (45/84) (Table 2). The mean household size at baseline was 4 individuals (± SD, min-max: 2, 1–8) with an average of 2 household members enrolled in the SHWS (± SD, min-max: 1, 1–5). The majority of participants had at least some high school education (48%, 40/84), with 8% (7/84) of participants having at least some middle school education, 17% of participants had an associate degree (14/84), 20% a bachelor’s degree (17/84), and 7% a master’s or professional degree (MD, PhD, MS, JD, or equivalent) (9/84). On average, participants were followed for 2.0 years (± SD, min-max: 0.5, 0.7–2.7). Over the course of follow-up, 51% (35/69) of participants lived in a household that reported changing their arsenic filter cartridge.

**Table 2:** Baseline demographics, baseline water use, and arsenic filter cartridge change over follow-up

	%	n	N
Participants			84
Households			50
Household size (household members)			
Mean ± SD (min–max)	4 ± 2 (1–8)		
Household members in SHWS			
Mean ± SD (min–max)	2 ± 1 (1–5)		
Age (years)			
Mean ± SD (min–max)	54 ± 19 (13–85)		
Sex			
Female	54%	45	84
Education			
Middle school	8%	7	84
High school	48%	40	84
Associate degree	17%	14	84
Bachelor’s degree	20%	17	84
Master’s degree or higher	7%	6	84
Duration of follow-up (years)			
Mean ± SD (min–max)	2.0 ± 0.5 (0.7–2.7)		
Exclusive safe water use			
Cooking	17%	14	84
Drinking	12%	10	84
Cooking and drinking	11%	9	84
Arsenic filter cartridge change	51%	35	69

n refers to the number of participants with the given characteristic. *SD* Standard deviation. Safe water defined as use of POU arsenic filter and/or bottled water

Baseline exclusive use of arsenic-safe water for drinking and cooking was low at 11% (9/84). Arsenic-safe water use at baseline was exclusively bottled water with the exception of one household that hauled water from a municipal source. Large increases in the exclusive use of arsenic-safe water were observed from baseline to follow-up. For drinking, exclusive use of arsenic-safe water increased from 12% (10/84) at baseline to 41% (31/75) at follow-up. For cooking, exclusive use of arsenic-safe water increased from 17% (14/84) at baseline to 48% (36/75) at follow-up. Overall exclusive use of arsenic-safe water for both drinking and cooking increased to 36% (27/75) at follow-up.

Behavioral determinants at baseline and follow-up and the change in score between timepoints are presented in Table 3. One behavioral determinant significantly differed by study arm at baseline (Supplementary Table 2). Some participants were not available when their household was initially enrolled and therefore may have been exposed to the intervention before being administered the behavioral determinant questionnaire at baseline. This is the most likely explanation for these results. Given the low frequency of this occurrence, we combined study arms for all analyses at baseline.

**Table 3:** Descriptive characteristics of behavioral determinants and knowledge items at baseline and follow-up and the change from baseline to follow-up

Statement	Behavioral determinant	Baseline (***N*** = 84)			Follow-up (***N*** = 75)			Change		
		Median	Mean	SD	Median	Mean	SD	Median	Mean	*p*-value
*How high or low are the chances of you getting health problems from arsenic if it is in your well water?*	Perceived vulnerability	0.50	0.61	0.31	0.75	0.67	0.32	0.25	0.06	0.177
*How high or low are the chances of someone in your household getting health problems from arsenic if it is in your well water?*	Perceived vulnerability	0.75	0.62	0.31	0.75	0.65	0.32	0.0	0.04	0.387
*How high or low are the chances of you getting health problems from arsenic if you drink water from an arsenic filter?*	Perceived vulnerability	0.25	0.20	0.23	0.25	0.25	0.30	0.0	0.05	0.137
*I have been drinking this water for a long time with no health problems, so I am not concerned about arsenic in my well water.*	Perceived vulnerability	**0.25**	**0.37**	**0.36**	**0.0**	**0.28**	**0.36**	**−0.25**	**−0.08**	**0.016**
*Because no one in my house has developed health problems from arsenic, I am not concerned about arsenic in my well water.*	Perceived vulnerability	0.25	0.32	0.35	0.0	0.29	0.35	−0.25	−0.03	0.212
*I can afford to fix my arsenic filter if it breaks.*	Perceived cost	0.75	0.61	0.36	0.75	0.66	0.39	0.0	0.05	0.088
*Of all the things I have to worry about, the arsenic filter is not at the top of my list.*	Competing Priorities	**0.25**	**0.42**	**0.37**	**0.75**	**0.56**	**0.40**	**0.50**	**0.14**	**0.027**
*If given the choice, I would prefer bottled water over water from an arsenic filter.*	User preferences	0.50	0.46	0.36	0.50	0.45	0.36	0.0	−0.01	0.495
*The tribal water system in my area has a safe level of arsenic.*	Perceived safety of tribal water system	**0.50**	**0.36**	**0.29**	**0.50**	**0.49**	**0.31**	**0.0**	**0.12**	**0.003**
*How many people in your community have arsenic in their wells?*	Perceived extent of contamination in community	0.75	0.60	0.26	0.50	0.62	0.30	−0.25	0.03	0.863
*How many people in your community with arsenic in their wells drink this water without filtration?*	Descriptive norm	**0.75**	**0.71**	**0.26**	**0.75**	**0.61**	**0.28**	**0.0**	**−0.10**	**0.003**
*How many people in your community with arsenic in their wells cook with this water without filtration?*	Descriptive norm	**0.75**	**0.75**	**0.23**	**0.75**	**0.64**	**0.28**	**0.0**	**−0.11**	**0.001**
*How many people in your community with arsenic in their wells use bottled water for drinking?*	Descriptive norm	**0.25**	**0.29**	**0.21**	**0.25**	**0.39**	**0.28**	**0.0**	**0.10**	**0.004**
*How many people in your community with arsenic in their wells use an arsenic filter for their drinking water?*	Descriptive norm	**0.25**	**0.16**	**0.18**	**0.25**	**0.30**	**0.27**	**0.0**	**0.14**	**0.001**
*How many people in your community with arsenic in their wells use an arsenic filter for their cooking water?*	Descriptive norm	**0.25**	**0.17**	**0.22**	**0.25**	**0.29**	**0.27**	**0.0**	**0.11**	**0.002**
*How much would people who are important to you approve or disapprove of you using water containing high arsenic for drinking?*	Injunctive norm	**0.25**	**0.22**	**0.26**	**0.00**	**0.11**	**0.22**	**−0.25**	**−0.11**	**0.012**
*How much would people who are important to you approve or disapprove of you using water containing high arsenic for cooking?*	Injunctive norm	**0.25**	**0.23**	**0.26**	**0.00**	**0.11**	**0.22**	**−0.25**	**−0.12**	**0.008**
*How sure are you that you can get drinking water with a safe level of arsenic?*	Self-efficacy	0.50	0.51	0.34	0.75	0.63	0.37	0.25	0.12	0.062
*How sure are you that you can get water for cooking with a safe level of arsenic?*	Self-efficacy	**0.50**	**0.49**	**0.35**	**0.75**	**0.66**	**0.34**	**0.25**	**0.17**	**0.004**
*How sure are you that you could find local resources to learn about arsenic in water?*	Self-efficacy	**0.25**	**0.40**	**0.36**	**0.50**	**0.54**	**0.36**	**0.25**	**0.13**	**0.016**
*How sure are you that local resources would help you resolve an arsenic-related problem with your private well?*	Self-efficacy	**0.25**	**0.36**	**0.36**	**0.50**	**0.49**	**0.36**	**0.25**	**0.13**	**0.016**
*How sure are you that you can use your arsenic filter faucet every time you need water for drinking in your home?*	Self-efficacy	**1.00**	**0.86**	**0.22**	**1.00**	**0.82**	**0.27**	**0.00**	**−0.04**	**0.034**
*How sure are you that you can use your arsenic filter faucet every time you need water for cooking in your home?*	Self-efficacy	1.00	0.86	0.22	1.00	0.84	0.26	0.00	−0.02	0.199
*How sure are you that you will be able to buy a new arsenic filter cartridge when needed?*	Self-efficacy	1.00	0.75	0.31	0.75	0.68	0.32	−0.25	−0.07	0.169
*How sure are you that you yourself can change your arsenic filter cartridge when needed?*	Self-efficacy	1.00	0.69	0.37	0.75	0.63	0.40	−0.25	−0.06	0.050
*How sure are you that you will be able to use your arsenic filter consistently over the next year?*	Self-efficacy	1.00	0.92	0.18	1.00	0.88	0.24	0.00	−0.04	0.051
*How sure are you that you will be able to use your filter consistently over the next five years?*	Self-efficacy	**1.00**	**0.85**	**0.22**	**1.00**	**0.80**	**0.30**	**0.00**	**−0.05**	**0.007**
*How committed do you feel to drinking water only from your arsenic filter faucet?*	Commitment strength	1.00	0.79	0.26	1.00	0.79	0.28	0.00	−0.01	0.808
*How committed do you feel to cooking with water only from your arsenic filter faucet?*	Commitment strength	1.00	0.82	0.25	1.00	0.81	0.28	0.00	0.00	0.655
*Overall arsenic knowledge*	Knowledge	0.71	0.66	0.28	0.71	0.65	0.29	0.00	−0.01	0.940
*Name two health conditions that can happen from arsenic exposure.*	Knowledge	0.50	0.54	0.44	0.50	0.54	0.41	0.00	0.00	0.819
*Name two tasks where it is OK to use water with high arsenic.*	Knowledge	1.00	0.61	0.44	1.00	0.61	0.45	0.00	−0.01	0.781
*Name two tasks where it is NOT OK to use water with high arsenic.*	Knowledge	1.00	0.76	0.35	1.00	0.72	0.40	0.00	−0.04	0.442
*How could you remove arsenic from drinking water?*	Knowledge	1.00	0.81	0.39	1.00	0.82	0.39	0.00	0.00	0.312

Behavioral determinants measured with Likert scale and rescaled for comparability. *P*-value measured with regression with generalized estimating equations to account for household level clustering. Change calculated as difference between follow-up and baseline (follow-up-baseline). *SD* Standard deviation. *P*-value < 0.05 indicated in bold. *P*-values unable to be calculated for behavioral determinants with no change on Likert scale

At baseline, most participants were committed to drinking and cooking with water from the POU arsenic filter faucet (drinking: mean: 0.79, SD: 0.26; cooking: mean: 0.82, SD: 0.25) (commitment strength). Most participants reported some disagreement to the statement “I have been drinking this water for a long time with no health problems, so I am not concerned about arsenic in my well water” (58%, mean: 0.37, SD: 0.36) (perceived vulnerability). The majority of participants were sure they could use their POU arsenic filter faucet every time for both drinking (mean: 0.86, SD: 0.22) and cooking (mean: 0.86, SD: 0.22) in the home (self-efficacy). At baseline, participants reported that most people in their community with arsenic in their wells drank (mean: 0.71, SD: 0.26) and cooked (mean: 0.75, SD: 0.23) with the contaminated water without filtration (descriptive norms). Participants also reported that only a few people in their community used bottled water for drinking (mean: 0.29, SD: 0.21) or used an POU arsenic filter for their drinking (mean: 0.16, SD: 0.18) or cooking water (mean: 0.17, SD: 0.22) (descriptive norms). The majority of participants mentioned disapproval among friends and family of using water containing high arsenic for both drinking (mean: 0.22, SD: 0.26) and cooking (mean: 0.23, SD: 0.26) (injunctive norms). Finally, participants reported some disagreement to the statement that of all the things they had to worry about, the arsenic filter was not on the top of their priority list (mean: 0.42, SD: 0.37) (competing priorities).

At baseline, 81% (68/84) of participants could correctly name at least one method to remove arsenic from water. The most commonly reported methods were filtration (correct) at 81% (68/84) of participants, boiling (incorrect) at 7% (6/84), and that arsenic cannot be removed from water (incorrect) at 4% (3/84) (Table 4). The majority of participants at baseline were able to correctly name two tasks where it is not okay to use arsenic contaminated water (62%, 52/84); a further 21% (18/84) correctly named one task and 17% (14/84) were unable to name any tasks. Drinking (correct) (81%, 68/84), cooking (correct) (61%, 51/84), and bathing/showering (incorrect) (6%, 5/84) were the most commonly mentioned tasks where it is not okay to use arsenic contaminated water. Similarly, 50% (42/84) of participants at baseline correctly named two tasks where it is okay to use arsenic contaminated water; a further 18% (15/84) of participants correctly named one task, and 32% (27/84) of participants could not name any tasks where it is okay to use arsenic contaminated water. The three most common responses to this item were bathing/showering (correct) (36%, 30/84), washing dishes (correct) (18%, 15/84), and laundry (correct) (15%, 13/84). Lastly, only 41% (34/83) of participants at baseline were able to correctly report two health conditions resulting from arsenic exposure; a further 21% (17/83) provided one correct response, and 39% (32/83) could not provide a correct health condition. Twenty-eight percent (23/83) of participants named cancer (correct), 13% (11/83) diabetes (correct), and 14% (12/83) heart disease (correct) as health conditions resulting from arsenic exposure.

**Table 4:** Arsenic knowledge themes at baseline and follow-up

**Name two health conditions that can happen from arsenic exposure:**				**Name two tasks where it is OK to use water with high arsenic:**			
**Major themes**	**Baseline % (*N*** **= 83)**	**Follow-up % (*N*** **= 70)**	**Response Category**	**Major themes**	**Baseline % (*N*** **= 84)**	**Follow-up % (*N*** **= 71)**	**Response Category**
Cancer	28% (23)	43% (30)	Correct	Bathing/showering	36% (30)	28% (20)	Correct
Do not know	33% (27)	29% (20)	Incorrect	Do not know	29% (24)	30% (21)	Incorrect
Diabetes	13% (11)	10% (7)	Correct	Washing dishes	18% (15)	31% (22)	Correct
Heart disease	14% (12)	9% (6)	Correct	Laundry	15% (13)	24% (17)	Correct
Death	6% (5)	11% (8)	Correct	Cleaning	10% (8)	13% (9)	Correct
**Name two tasks where it is NOT OK to use water with high arsenic:**				**How could you remove arsenic from drinking water?**			
**Major themes**	**Baseline % (*N*** **= 84)**	**Follow-up % (*N*** **= 71)**	**Response Category**	**Major themes**	**Baseline % (*N*** **= 84)**	**Follow-up % (*N*** **= 71)**	**Response Category**
Drinking	81% (68)	72% (51)	Correct	Filter	81% (68)	82% (58)	Correct
Cooking	61% (51)	65% (46)	Correct	Boiling	7% (6)	18% (13)	Incorrect
Do not know	17% (14)	17% (12)	Incorrect	Do not know	15% (13)	6% (4)	Incorrect
Bathing/showering	6% (5)	4% (3)	Incorrect	Arsenic cannot be removed from water	4% (3)	7% (5)	Incorrect
Water plants	5% (4)	0% (0)	Incorrect	Use alternative source (change well)	2% (2)	0% (0)	Correct

N refers to the number of participants. Participants were allowed two responses to each question. The 5 most common themes are reported for each item, regardless of correctness. “Do not know” refers to those who replied that they did not know for all responses to the given question

All changes in behavioral determinants and knowledge items from baseline to follow-up were in the hypothesized direction. Concern about future personal health problems from long-term arsenic exposure (*p* = 0.016) (perceived vulnerability) increased from baseline to follow-up (Table 3). Self-efficacy to obtain arsenic-safe water for drinking (*p* = 0.062) and cooking (*p* = 0.004) also increased over this time. Participants’ perceptions of the proportion of community members drinking and cooking with arsenic-safe water significantly increased from baseline to follow-up (drinking (*p* = 0.001) and cooking (*p* = 0.002)), as well as a significant increase in the perceived proportion of community members that used bottled water for drinking (*p* = 0.004) (descriptive norms). Consistent with this, during the study period there was a significant decrease in the perceived use of arsenic-unsafe well water for drinking in the community (*p* = 0.003) (descriptive norms). There was also a significant increase in participant’s perceived disapproval of their peers in using arsenic-safe water for drinking and cooking from baseline to follow-up (drinking (*p* = 0.012) and cooking (*p* = 0.008)) (injunctive norms). There were no significant changes in commitment strength or arsenic knowledge from baseline to follow-up. Changes from baseline to follow-up for each study arm are provided in Supplementary Table 3.

Higher commitment to exclusively drink and cook with water from the POU arsenic filter faucet at baseline was associated with significantly higher odds of exclusive use of arsenic-safe water for cooking (OR: 15.90, 95% CI: 1.33–189.52) and drinking (OR: 32.57, 95% CI: 1.42–746.70) at follow-up (Table 5). Greater concern about arsenic even if someone had been drinking arsenic contaminated water for a long time without health related problems (OR: 0.22, 95% CI: 0.06–0.81 (lower score = greater concern about arsenic)) (perceived vulnerability), greater confidence in one’s ability to find local resources to learn about arsenic in water (OR: 5.19, 95% CI: 1.48–18.21) (self-efficacy), and higher confidence in the usefulness of local resources to resolve an arsenic-related problem with a private well (OR: 3.11, 95% CI: 1.11–8.71) (self-efficacy) at baseline were significantly associated with exclusive use of arsenic-safe water at follow-up. Higher confidence in one’s ability to use the POU arsenic filter faucet every time for drinking water in the home at baseline was significantly associated with exclusive use of arsenic-safe water for drinking at follow-up (OR: 9.17, 95% CI: 1.15–73.35) (self-efficacy). Higher agreement that the POU arsenic filter faucet was not a priority compared to other worries at baseline was also associated with exclusive use of arsenic-safe water for drinking and cooking at follow-up (OR: 0.23, 95% CI: 0.06–0.94). Providing a correct response to the knowledge item “How could you remove arsenic from drinking water?” at baseline was significantly associated with exclusive use of arsenic-safe water for cooking at follow-up (OR: 3.52, 95% CI: 1.07–11.58). Sex, education, and age were not significant for any of the three exclusive use outcomes assessed (Supplementary Table 4).

**Table 5:** Influence of behavioral determinants and knowledge items at baseline on follow-up exclusive use of arsenic-safe water

Statement	Behavioral determinant	Exclusive use of arsenic-safe water								
		Cooking			Drinking			Cooking and drinking		
		OR	95% CI	*p*-value	OR	95% CI	*p*-value	OR	95% CI	*p*-value
*How high or low are the chances of you getting health problems from arsenic if it is in your well water?*	Perceived vulnerability	1.50	0.39, 5.77	0.559	0.70	0.26, 1.89	0.483	1.16	0.35, 3.89	0.810
*How high or low are the chances of someone in your household getting health problems from arsenic if it is in your well water?*	Perceived vulnerability	1.64	0.43, 6.22	0.465	0.72	0.28, 1.87	0.499	1.26	0.38, 4.23	0.704
*How high or low are the chances of you getting health problems from arsenic if you drink water from an arsenic filter?*	Perceived vulnerability	0.28	0.04, 1.85	0.184	0.40	0.06, 2.51	0.325	0.48	0.07, 3.52	0.470
*I have been drinking this water for a long time with no health problems, so I am not concerned about arsenic in my well water.*	Perceived vulnerability	**0.26**	**0.07, 0.98**	**0.047**	0.36	0.11, 1.12	0.078	**0.22**	**0.06, 0.81**	**0.024**
*Because no one in my house has developed health problems from arsenic, I am not concerned about arsenic in my well water.*	Perceived vulnerability	**0.20**	**0.06, 0.65**	**0.008**	**0.34**	**0.13, 0.86**	**0.024**	**0.21**	**0.07, 0.62**	**0.005**
*I can afford to fix my arsenic filter if it breaks.*	Perceived cost	2.67	0.70, 10.14	0.149	2.29	0.76, 6.96	0.143	2.02	0.59, 6.97	0.264
*Of all the things I have to worry about, the arsenic filter is not at the top of my list.*	Competing Priorities	0.27	0.07, 1.03	0.055	0.36	0.11, 1.26	0.112	**0.23**	**0.06, 0.94**	**0.041**
*If given the choice, I would prefer bottled water over water from an arsenic filter.*	User Preferences	0.26	0.06, 1.04	0.057	0.49	0.16, 1.54	0.225	0.27	0.07, 1.04	0.057
*The tribal water system in my area has a safe level of arsenic.*	Perceived safety of tribal water system	1.21	0.21, 7.10	0.832	0.50	0.12, 2.00	0.329	0.78	0.18, 3.41	0.740
*How many people in your community have arsenic in their wells?*	Perceived extent of contamination in community	0.78	0.12, 5.03	0.798	0.74	0.23, 2.41	0.617	0.51	0.12, 2.15	0.356
*How many people in your community with arsenic in their wells drink this water without filtration?*	Descriptive norm				0.86	0.23, 3.17	0.819	1.10	0.28, 4.34	0.892
*How many people in your community with arsenic in their wells cook with this water without filtration?*	Descriptive norm	0.73	0.10, 5.16	0.750				0.61	0.11, 3.35	0.571
*How many people in your community with arsenic in their wells use bottled water for drinking?*	Descriptive norm				1.24	0.24, 6.35	0.800	1.07	0.19, 6.04	0.937
*How many people in your community with arsenic in their wells use an arsenic filter for their drinking water?*	Descriptive norm				1.16	0.34, 4.00	0.816	1.20	0.23, 6.20	0.826
*How many people in your community with arsenic in their wells use an arsenic filter for their cooking water?*	Descriptive norm	0.69	0.15, 3.11	0.624				1.06	0.33, 3.35	0.926
*How much would people who are important to you approve or disapprove of you using water containing high arsenic for drinking?*	Injunctive norm				0.69	0.13, 3.48	0.648	0.22	0.04, 1.23	0.085
*How much would people who are important to you approve or disapprove of you using water containing high arsenic for cooking?*	Injunctive norm	0.29	0.05, 1.55	0.148				0.36	0.06, 2.19	0.269
*How sure are you that you can get drinking water with a safe level of arsenic?*	Self-efficacy				1.10	0.35, 3.44	0.864	1.35	0.38, 4.88	0.643
*How sure are you that you can get water for cooking with a safe level of arsenic?*	Self-efficacy	1.12	0.33, 3.74	0.858				1.03	0.32, 3.38	0.958
*How sure are you that you could find local resources to learn about arsenic in water?*	Self-efficacy	**9.60**	**2.76, 33.35**	**0.000**	2.57	0.82, 8.04	0.104	**5.19**	**1.48, 18.21**	**0.010**
*How sure are you that local resources would help you resolve an arsenic-related problem with your private well?*	Self-efficacy	**3.58**	**1.22, 10.50**	**0.022**	2.06	0.84, 5.06	0.114	**3.11**	**1.11, 8.71**	**0.031**
*How sure are you that you can use your arsenic filter faucet every time you need water for drinking in your home?*	Self-efficacy				**9.17**	**1.15, 73.35**	**0.037**	9.35	0.82, 107.19	0.073
*How sure are you that you can use your arsenic filter faucet every time you need water for cooking in your home?*	Self-efficacy	2.78	0.32, 23.93	0.353				5.85	0.56, 61.13	0.140
*How sure are you that you will be able to buy a new arsenic filter cartridge when needed?*	Self-efficacy	3.19	0.77, 13.21	0.110	**4.44**	**1.38, 14.25**	**0.012**	3.54	0.94, 13.37	0.062
*How sure are you that you yourself can change your arsenic filter cartridge when needed?*	Self-efficacy	**4.59**	**1.43, 14.77**	**0.011**	1.74	0.44, 6.92	0.435	2.22	0.54, 9.19	0.270
*How sure are you that you will be able to use your arsenic filter consistently over the next year?*	Self-efficacy	26.98	0.62, 1181.91	0.088	21.29	0.47, 965.67	0.116	19.93	0.23, 1750.44	0.190
*How sure are you that you will be able to use your filter consistently over the next five years?*	Self-efficacy	**16.59**	**1.43, 192.12**	**0.025**	5.56	0.71, 43.29	0.101	7.81	0.64, 94.81	0.107
*How committed do you feel to drinking water only from your arsenic filter faucet?*	Commitment strength				**9.38**	**1.02, 86.55**	**0.048**	**32.57**	**1.42, 746.70**	**0.029**
*How committed do you feel to cooking with water only from your arsenic filter faucet?*	Commitment strength	**8.53**	**1.25, 58.14**	**0.029**				**15.90**	**1.33, 189.52**	**0.029**
*Overall arsenic knowledge*	Knowledge	5.04	0.87, 29.13	0.071	2.43	0.34, 17.25	0.376	2.38	0.34, 16.82	0.386
*Name two health conditions that can happen from arsenic exposure?*	Knowledge	0.93	0.35, 2.48	0.882	0.68	0.26, 1.77	0.427	0.74	0.28, 1.98	0.554
*Name two tasks where it is OK to use water with high arsenic?*	Knowledge	2.24	0.72, 7.02	0.167	2.91	0.82, 10.30	0.097	2.08	0.52, 8.29	0.300
*Name two tasks where it is NOT OK to use water with high arsenic?*	Knowledge	5.33	0.88, 32.19	0.068	2.39	0.50, 11.47	0.280	2.70	0.49, 14.84	0.253
*How could you remove arsenic from drinking water?*	Knowledge	**3.52**	**1.07, 11.58**	**0.039**	1.44	0.46, 4.51	0.536	1.81	0.47, 6.98	0.389

Odds ratios calculated with regression with generalized estimating equations to account for household level clustering. *P*-value < 0.05 indicated in bold. Associations not calculated for variables assessing a different type of water use

Increased commitment to cook only with water from the POU arsenic filter faucet from baseline to follow-up was significantly associated with the exclusive use of arsenic-safe water for both cooking and drinking (OR: 4.24, 95% CI: 1.15–15.66) and for cooking only (OR: 8.89, 95% CI: 1.84–43.06) at follow-up (Table 6). Increased perceived vulnerability from baseline to follow-up related to the chances of a household member developing health problems from arsenic exposure in well water was also significantly associated with exclusive use of arsenic-safe water for drinking at follow-up (OR: 3.44, 95% CI: 1.04–11.3370). Additionally, increased self-efficacy related to the use the POU arsenic filter faucet every time for cooking in the home was significantly associated with exclusive use of arsenic-safe water for cooking at follow-up (OR: 11.5, 95% CI: 1.41, 92.79).

**Table 6:** Influence of change in behavioral determinants from baseline to follow-up on use of exclusive use of arsenic-safe water at follow-up

Statement	Behavioral determinant	Exclusive use of arsenic-safe water								
		Cooking			Drinking			Cooking and drinking		
		OR	95% CI	*p*-value	OR	95% CI	*p*-value	OR	95% CI	*p*-value
*How high or low are the chances of you getting health problems from arsenic if it is in your well water?*	Perceived vulnerability	1.21	0.33, 4.49	0.773	3.10	0.97, 9.85	0.055	2.04	0.42, 9.92	0.375
*How high or low are the chances of someone in your household getting health problems from arsenic if it is in your well water?*	Perceived vulnerability	1.57	0.43, 5.73	0.495	3.42	1.04, 11.33	0.044	2.16	0.43, 10.89	0.352
*How high or low are the chances of you getting health problems from arsenic if you drink water from an arsenic filter?*	Perceived vulnerability	0.91	0.22, 3.72	0.895	1.03	0.32, 3.28	0.967	0.62	0.13, 2.87	0.540
*I have been drinking this water for a long time with no health problems, so I am not concerned about arsenic in my well water.*	Perceived vulnerability	0.19	0.03, 1.15	0.071	1.21	0.32, 4.57	0.776	0.75	0.19, 2.92	0.683
*Because no one in my house has developed health problems from arsenic, I am not concerned about arsenic in my well water.*	Perceived vulnerability	1.04	0.25, 4.36	0.96	1.63	0.63, 4.27	0.317	1.01	0.35, 2.94	0.979
*I can afford to fix my arsenic filter if it breaks.*	Perceived cost	1.35	0.45, 4.00	0.592	0.97	0.32, 2.90	0.950	1.34	0.45, 3.96	0.595
*Of all the things I have to worry about, the arsenic filter is not at the top of my list.*	Competing Priorities	1.46	0.63, 3.43	0.379	1.22	0.57, 2.59	0.605	1.50	0.63, 3.56	0.355
*If given the choice, I would prefer bottled water over water from an arsenic filter.*	User preferences	0.95	0.30, 2.97	0.926	1.08	0.51, 2.30	0.838	1.04	0.42, 2.58	0.934
*The tribal water system in my area has a safe level of arsenic.*	Perceived safety of tribal water system	0.96	0.29, 3.14	0.940	1.96	0.87, 4.40	0.102	1.88	0.78, 4.52	0.157
*How many people in your community have arsenic in their wells?*	Perceived extent of contamination in community	1.43	0.32, 6.26	0.638	2.35	0.79, 7.03	0.126	2.54	0.78, 8.21	0.120
*How many people in your community with arsenic in their wells drink this water without filtration?*	Descriptive norm				3.42	0.85, 13.74	0.083	1.89	0.51, 6.94	0.34
*How many people in your community with arsenic in their wells cook with this water without filtration?*	Descriptive norm	2.56	0.49, 13.46	0.266				1.98	0.62, 6.29	0.248
*How many people in your community with arsenic in their wells use bottled water for drinking?*	Descriptive norm				1.96	0.34, 11.28	0.449	2.13	0.37, 12.26	0.398
*How many people in your community with arsenic in their wells use an arsenic filter for their drinking water?*	Descriptive norm				1.31	0.30, 5.61	0.720	1.98	0.45, 8.61	0.364
*How many people in your community with arsenic in their wells use an arsenic filter for their cooking water?*	Descriptive norm	2.65	0.53, 13.25	0.236				1.97	0.58, 6.71	0.277
*How much would people who are important to you approve or disapprove of you using water containing high arsenic for drinking?*	Injunctive norm				1.39	0.29, 6.63	0.683	2.08	0.43, 9.94	0.361
*How much would people who are important to you approve or disapprove of you using water containing high arsenic for cooking?*	Injunctive norm	3.34	0.74, 15.11	0.117				1.86	0.37, 9.31	0.448
*How sure are you that you can get drinking water with a safe level of arsenic?*	Self-efficacy				2.23	0.85, 5.83	0.103	2.83	0.91, 8.82	0.073
*How sure are you that you can get water for cooking with a safe level of arsenic?*	Self-efficacy	2.18	0.79, 5.98	0.132				2.25	0.87, 5.79	0.094
*How sure are you that you could find local resources to learn about arsenic in water?*	Self-efficacy	0.53	0.16, 1.73	0.289	0.46	0.14, 1.48	0.191	0.58	0.17, 2.00	0.386
*How sure are you that local resources would help you resolve an arsenic-related problem with your private well?*	Self-efficacy	0.67	0.24, 1.88	0.450	1.31	0.52, 3.31	0.575	1.07	0.39, 2.94	0.888
*How sure are you that you can use your arsenic filter faucet every time you need water for drinking in your home?*	Self-efficacy				1.47	0.28, 7.79	0.648	3.42	0.47, 24.86	0.224
*How sure are you that you can use your arsenic filter faucet every time you need water for cooking in your home?*	Self-efficacy	11.45	1.41, 92.79	0.022				3.08	0.47, 20.08	0.239
*How sure are you that you will be able to buy a new arsenic filter cartridge when needed?*	Self-efficacy	2.96	0.58, 15.03	0.191	1.94	0.51, 7.39	0.331	3.35	0.85, 13.27	0.085
*How sure are you that you yourself can change your arsenic filter cartridge when needed?*	Self-efficacy	0.95	0.41, 2.21	0.910	1.01	0.33, 3.03	0.991	0.92	0.30, 2.84	0.889
*How sure are you that you will be able to use your arsenic filter consistently over the next year?*	Self-efficacy	6.97	0.73, 66.98	0.092	1.81	0.47, 6.98	0.391	3.09	0.71, 13.43	0.133
*How sure are you that you will be able to use your filter consistently over the next five years?*	Self-efficacy	2.62	0.53, 12.87	0.236	2.38	0.59, 9.64	0.225	2.91	0.70, 12.07	0.142
*How committed do you feel to drinking water only from your arsenic filter faucet?*	Commitment strength				1.13	0.23, 5.49	0.876	1.56	0.30, 8.21	0.597
*How committed do you feel to cooking with water only from your arsenic filter faucet?*	Commitment strength	**8.89**	**1.84, 43.06**	**0.007**				**4.24**	**1.15, 15.66**	**0.030**
*Arsenic knowledge*	Knowledge	0.95	0.19, 4.81	0.949	0.50	0.10, 2.55	0.403	0.53	0.10, 2.82	0.460
*Name two health conditions that can happen from arsenic exposure.*	Knowledge	1.05	0.38, 2.88	0.920	1.35	0.61, 2.98	0.454	0.83	0.37, 1.87	0.662
*Name two tasks where it is OK to use water with high arsenic.*	Knowledge	1.32	0.56, 3.10	0.524	0.56	0.24, 1.30	0.174	0.68	0.29, 1.61	0.380
*Name two tasks where it is NOT OK to use water with high arsenic.*	Knowledge	0.70	0.23, 2.16	0.535	0.59	0.20, 1.74	0.338	0.76	0.27, 2.12	0.602
*How could you remove arsenic from drinking water.*	Knowledge	0.51	0.20, 1.33	0.170	1.39	0.50, 3.87	0.526	1.38	0.49, 3.91	0.539

*P*-values calculated with regression with generalized estimating equations to account for household level clustering. *P*-value < 0.05 indicated in bold. Associations not calculated for variables assessing a different type of water use. OR = Odds ratio

Perceived vulnerability, self-efficacy, and competing life priorities were significantly changed with overall SHWS program delivery from baseline to follow-up *and* were associated with the exclusive use of arsenic-safe water at follow-up. Concern about arsenic even if someone had been drinking arsenic contaminated water for a long time without health related problems  (perceived vulnerability) increased during the SHWS program period, and this change led to increases in exclusive use of arsenic-safe water for both drinking and cooking at follow-up. The ability to find local resources to learn about arsenic in water (self-efficacy) and the belief that these resources would be helpful to resolve arsenic-related well problems (self-efficacy) significantly increased during the study period, and this was associated with exclusive use of arsenic-safe water for both cooking and drinking at follow-up. Finally, participants were less worried about their POU arsenic filter faucet compared to other priorities at baseline compared to follow-up, and this was associated with exclusive use of arsenic-safe water for both cooking and drinking at follow-up.

Greater concern about arsenic even if someone had been drinking arsenic contaminated water for a long time without health-related problems (OR: 7.79, 95% CI: 1.17–51.98) (perceived vulnerability) or a household member had been drinking arsenic contaminated water for a long time without health-related problems (OR: 11.41, 95% CI: 1.75–74.44) (perceived vulnerability) at baseline were both associated with a higher likelihood of changing the arsenic filter cartridge after installation (Table 7). Higher baseline self-efficacy in the perceived ability to obtain water with a safe level of arsenic for drinking (OR: 6.22, 95% CI: 1.33–29.07) and for cooking (OR: 10.65, 95% CI: 2.48–45.68) was also associated with a higher likelihood of changing the arsenic filter cartridge after installation. There was no association between the demographic variables assessed and arsenic filter cartridge change (Supplementary Table 5).

**Table 7:** Influence of behavioral determinants at baseline on arsenic filter cartridge change after installation

Statement	Behavioral determinant	Arsenic filter cartridge change after installation		
		OR	95% CI	*p*-value
*How high or low are the chances of you getting health problems from arsenic if it is in your well water?*	Perceived vulnerability	**7.79**	**1.17, 51.98**	**0.034**
*How high or low are the chances of someone in your household getting health problems from arsenic if it is in your well water?*	Perceived vulnerability	**11.41**	**1.75, 74.44**	**0.011**
*How high or low are the chances of you getting health problems from arsenic if you drink water from an arsenic filter?*	Perceived vulnerability	0.70	0.09, 5.53	0.735
*I have been drinking this water for a long time with no health problems, so I am not concerned about arsenic in my well water.*	Perceived vulnerability	0.36	0.08, 1.68	0.195
*Because no one in my house has developed health problems from arsenic, I am not concerned about arsenic in my well water.*	Perceived vulnerability	0.30	0.08, 1.16	0.081
*I can afford to fix my arsenic filter if it breaks.*	Perceived cost	0.87	0.19, 4.08	0.859
*Of all the things I have to worry about, the arsenic filter is not at the top of my list.*	Competing Priorities	0.29	0.06, 1.35	0.116
*If given the choice, I would prefer bottled water over water from an arsenic filter.*	User preferences	1.32	0.32, 5.43	0.703
*The tribal water system in my area has a safe level of arsenic.*	Perceived safety of tribal water system	1.29	0.22, 7.69	0.781
*How many people in your community have arsenic in their wells?*	Perceived extent of contamination in community	0.83	0.10, 6.67	0.857
*How many people in your community with arsenic in their wells drink this water without filtration?*	Descriptive norm	0.17	0.03, 1.01	0.051
*How many people in your community with arsenic in their wells cook with this water without filtration?*	Descriptive norm	0.15	0.02, 1.03	0.054
*How many people in your community with arsenic in their wells use bottled water for drinking?*	Descriptive norm	2.86	0.34, 24.29	0.336
*How many people in your community with arsenic in their wells use an arsenic filter for their drinking water?*	Descriptive norm	3.65	0.17, 79.91	0.410
*How many people in your community with arsenic in their wells use an arsenic filter for their cooking water?*	Descriptive norm	1.57	0.25, 9.84	0.628
*How much would people who are important to you approve or disapprove of you using water containing high arsenic for drinking?*	Injunctive norm	0.80	0.10, 6.74	0.84
*How much would people who are important to you approve or disapprove of you using water containing high arsenic for cooking?*	Injunctive norm	1.40	0.19, 10.06	0.741
*How sure are you that you can get drinking water with a safe level of arsenic?*	Self-efficacy	**6.22**	**1.33, 29.07**	**0.020**
*How sure are you that you can get water for cooking with a safe level of arsenic?*	Self-efficacy	**10.65**	**2.48, 45.68**	**0.001**
*How sure are you that you could find local resources to learn about arsenic in water?*	Self-efficacy	1.42	0.31, 6.47	0.654
*How sure are you that local resources would help you resolve an arsenic-related problem with your private well?*	Self-efficacy	7.69	1.49, 39.64	0.015
*How sure are you that you can use your arsenic filter faucet every time you need water for drinking in your home?*	Self-efficacy	0.94	0.10, 8.43	0.955
*How sure are you that you can use your arsenic filter faucet every time you need water for cooking in your home?*	Self-efficacy	2.43	0.25, 23.41	0.442
*How sure are you that you will be able to buy a new arsenic filter cartridge when needed?*	Self-efficacy	4.22	0.60, 29.73	0.148
*How sure are you that you yourself can change your arsenic filter cartridge when needed?*	Self-efficacy	3.12	0.87, 11.23	0.082
*How sure are you that you will be able to use your arsenic filter consistently over the next year?*	Self-efficacy	6.82	0.35, 133.44	0.210
*How sure are you that you will be able to use your filter consistently over the next five years?*	Self-efficacy	1.68	0.18, 15.64	0.650
*How committed do you feel to drinking water only from your arsenic filter faucet?*	Commitment strength	3.02	0.35, 26.32	0.317
*How committed do you feel to cooking with water only from your arsenic filter faucet?*	Commitment strength	7.86	0.76, 81.41	0.084
*Overall arsenic knowledge*	Knowledge	2.66	0.45, 15.61	0.278
*Name two health conditions that can happen from arsenic exposure.*	Knowledge	2.48	0.83, 7.43	0.105
*Name two tasks where it is OK to use water with high arsenic.*	Knowledge	0.79	0.23, 2.72	0.711
*Name two tasks where it is NOT OK to use water with high arsenic.*	Knowledge	2.72	0.70, 10.54	0.148
*How could you remove arsenic from drinking water.*	Knowledge	1.85	0.53, 6.38	0.332

Odds ratios (OR) calculated with regression with generalized estimating equations to account for household level clustering. *P*-value < 0.05 indicated in bold

## Discussion

This study investigated behavioral determinants associated with exclusive use of arsenic-safe water and changing ones arsenic filter cartridge for households receiving the community-led SHWS arsenic mitigation program. The SHWS program significantly increased perceived vulnerability to arsenic exposure, and self-efficacy, descriptive norms, and injunctive norms related to the use of arsenic-safe water. Increased perceived vulnerability, self-efficacy, and commitment strength to using the POU arsenic filter faucet after SHWS program delivery was associated with higher exclusive use of arsenic-safe water at follow-up. Perceived vulnerability and self-efficacy were also associated with changing one’s arsenic filter cartridge during the follow-up period. These results suggest that the community-led SHWS program was effective in changing the targeted behavioral determinants of the use of arsenic-safe water and changing ones arsenic filter cartridge, and thereby increased these behaviors. This study demonstrates the effectiveness of theory driven community-led intervention approaches to reduce arsenic exposure. These findings complement those from George et al. and Zacher et al., which found that delivery of the SHWS intervention significantly reduced arsenic exposure (George et al., submitted; [52]). These behavioral determinants of arsenic-safe water use and changing ones arsenic filter cartridge will be targeted in scaling efforts for this arsenic mitigation program. 

Commitment strength to only drink and cook using water from the POU arsenic filter faucet was the behavioral determinant most strongly associated with the exclusive use of arsenic-safe water for both cooking and drinking. Numerous studies have highlighted the connection between commitment strength and behaviors to protect against water arsenic exposure, both in the United States and internationally [25–29, 53]. Commitment strength was high at baseline, with no significant  change in reported commitment strength between baseline and follow-up with SHWS program delivery. It is possible that those who participated in the SHWS did so because they were already committed to reducing their arsenic exposure. While overall commitment strength did not change significantly between baseline and follow-up, our findings indicate that an increase in commitment from baseline to follow-up to cook only with water from the POU arsenic filter faucet was associated with a higher likelihood of exclusive use of arsenic-safe water at follow-up. This finding suggests that for the participants whose commitment to only cook with arsenic-safe water increased, this increase was an important contributor to their arsenic-safe water use. Future studies assessing approaches for arsenic mitigation should evaluate the impact of arsenic interventions on commitment strength.

Perceived vulnerability to the health effects of arsenic exposure increased with SHWS delivery and was associated with exclusive use of arsenic-safe water for both cooking and drinking. This finding is consistent with previous studies conducted both in the United States and in Bangladesh that assessed arsenic mitigation [25, 27, 54] and use of arsenic mitigation options [25, 27]. In one study in New Jersey examining health protective behaviors to reduce arsenic exposure, perceived susceptibility to arsenic exposure was a significant predictor of the use of an arsenic mitigation option [27]. These findings highlight the importance of changes in perceived vulnerability for sustained use of arsenic-safe water in our program setting, and indicate that our health communication on the health implications of long-term arsenic exposure increased perceived vulnerability on the health effects of arsenic within our partner communities, and subsequently increased the use of arsenic-safe water.

Self-efficacy at baseline was strongly associated with exclusive use of arsenic-safe water for both cooking and drinking at follow-up. Our findings suggest that those who are confident in their ability to use local resources to resolve an arsenic-related problem with their private well are more likely to perform protective behaviors to reduce their arsenic exposure. This is consistent with results of previous studies that have highlighted the importance of self-efficacy in water arsenic protective behaviors [25, 27, 55, 56]. Severson et al. found that among a survey of homeowners with high-arsenic in their wells, accessing arsenic information increased protective behaviors [24]. This highlights the importance of our finding that when participants are confident in their ability to access local resources about arsenic, they are more likely to exclusively use arsenic-safe water.

The SHWS did not significantly increase arsenic knowledge between baseline and follow-up. However, baseline knowledge of how to remove arsenic from drinking water was associated with higher exclusive use of arsenic-safe water. One potential explanation for this finding is that because well water arsenic testing occurred prior to study enrollment, participants had already received some information regarding water arsenic levels, how to remove arsenic, and the possible health related effects of arsenic exposure. A few participants may have also been exposed to the intervention prior to their baseline questionnaire if they were not available when the initial household enrollment occurred. Even after the SHWS program was delivered, 18% of participants at follow-up still reported boiling could remove arsenic, and 17% did not know which household tasks could be safely performed with arsenic-safe water. Future SHWS health communication should work to further improve understanding of how to remove elevated arsenic from water and which tasks are safe to perform with contaminated water.

Participants who were less worried about their POU arsenic filter compared to other concerns at baseline were more likely to report exclusive use of arsenic-safe water at follow-up. While initially unexpected, it is possible that these individuals placed higher trust in the POU arsenic filter faucet to provide arsenic-safe water, and thus devoted less time worrying about their filters. Many individuals in our study face numerous challenges in their daily lives apart from arsenic contamination of well water [44], thereby providing the arsenic filter may have enabled them to spend less time worrying about arsenic in their water. 

None of the demographic factors assessed (age, sex, and education) predicted exclusive use of arsenic-safe water. Results from previous studies on the influence of demographic factors on arsenic treatment and mitigation and well testing for arsenic have yielded varying results [23, 25, 57–59]. In one study in central Maine, use of an arsenic treatment system was associated with higher education and income, while drinking bottled water was associated with lower education and income [25]. In another study in rural Nevada, lower education was associated with the decision to treat water for arsenic [23]. Finally, among families with children in rural Montana and Washington utilizing a non-municipal water source and demographic variables including age, education and income were similar between those who choose to take precautions against arsenic contaminated water and those that did not [59].

Changing the POU arsenic filter cartridge over the study period was associated with higher baseline self-efficacy in the ability to obtain arsenic-safe water for drinking and cooking and higher perceived vulnerability in the health effects from arsenic at both the personal and household levels. Changing one’s arsenic filter cartridge is an integral aspect of the SHWS program. If the POU arsenic filter is not changed as recommended, it may not reduce arsenic concentrations below 10 μg/L. Participants may therefore be unknowingly consuming arsenic-unsafe water despite using the arsenic faucet for drinking and cooking. To the authors knowledge, no study has specifically assessed prospectively behavioral determinants associated with changing or maintaining a POU arsenic filter. In one cross-sectional survey conducted in New Jersey among those treating water for arsenic, level of commitment and signing a service agreement were associated with treatment maintenance in all models tested [27]. Contrary to our results, perceived susceptibility was not associated with treatment maintenance. Our finding that higher perceived vulnerability of the health effects of arsenic influenced changing one’s filter cartridge may indicate that those who saw consumption of arsenic-safe water as a greater risk were more committed to overcome challenges in changing the filter cartridge. Future studies should investigate behavioral determinants of maintaining arsenic mitigation options over time in other settings globally so that interventions can target these behavioral determinants. 

This study has some strengths and limitations. The first strength is that this study reports findings from the first RCT evaluating an arsenic mitigation program to reduce water arsenic exposure in North America. Second, this study presented the unique opportunitie to prospectively assess behavioral determinants of the use of arsenic-safe water and changing ones POU arsenic filter cartridge and the impact of the community-led SHWS program on these behavioral determinants. Another strength of this study is information on both exclusive use of arsenic-safe water for drinking and cooking, and changing one’s filter cartridge for arsenic. Previous intervention studies only focused on behavioral determinants of arsenic-safe water. The first limitation is the small sample size due to study enrollment being halted because of the COVID-19 pandemic which limited the ability to assess differences between study arms. Second, COVID-19 impacted study visit timelines and in-person visits.

## Conclusions

Millions are exposed to unsafe levels of arsenic in water used for drinking and cooking globally. The community-led SHWS arsenic mitigation program conducted a theory-driven approach for intervention development and evaluation that allowed for behavioral determinants to be identified that were associated with the use of arsenic-safe water and changing one’s arsenic filter cartridge. This program increased perceived vulnerability to arsenic exposure and self-efficacy to obtain arsenic-safe water and these changes were associated with exclusive use of arsenic-safe water. Perceived vulnerability to arsenic exposure and self-efficacy were associated with changing one’s arsenic filter cartridge before the final follow-up. These results demonstrate that theory-driven, context-specific formative research can influence behavior change interventions to reduce water arsenic exposure. The SHWS can serve as a model for the design of theory-driven intervention approaches that engage communities to reduce their arsenic exposure.

## Supplementary Information

**Additional file 1: Supplementary Table 1.** Likert question response options. **Supplementary Table 2.** Differences in behavioral determinants at baseline by study arm. **Supplementary Table 3.** Baseline, follow-up, and change over study period for behavioral determinants by study arm. **Supplementary Table 4.** Influence of demographic factors on follow-up use of arsenic-safe water. **Supplementary Table 5.** Influence of demographic factors on arsenic filter cartridge change.

## Data Availability

The de-identified data supporting the conclusions of this article are available from the corresponding author upon reasonable request.

## Abbreviations

POU: Point-of-use
SHWS: Strong Heart Water Study
EPA: Environmental Protection Agency
MCL: Maximum contaminant level
POE: Point-of-entry
RCTs: Randomized controlled trials
RANAS: Risks, Attitudes, Norms, Abilities, and Self-regulation model of health behavior change
WASH: Water, sanitation, and hygiene
IHS: Indian Health Service
MBIRI: Missouri Breaks Industries Research, Inc.
ICP-MS: Inductively coupled plasma mass spectrometry
mHealth: Mobile health
GEE: Generalized estimating equations
