# Supplementary material for: Behavioral determinants of arsenic-safe water use among Great Plains Indian Nation private well users: results from the Community-Led Strong Heart Water Study Arsenic Mitigation Program
Source: Environ Health. 2023 May 15;22:42. doi: 10.1186/s12940-023-00965-0 (PMC10183246; doi:10.1186/s12940-023-00965-0)
Supplement: Supplementary file 1 — Additional file 1: Supplementary Table 1. Likert question response options. Supplementary Table 2. Differences in behavioral determinants at baseline by study arm. Supplementary Table 3. Baseline, follow-up, and change over study period for behavioral determinants by study arm. Supplementary Table 4. Influence of demographic factors on follow-up use of arsenic-safe water. Supplementary Table 5. Influence of demographic factors on arsenic filter cartridge change. [file 12940_2023_965_MOESM1_ESM.docx]

| **Supplementary Table 1.** Likert question response options | | | | | | |  |
| --- | --- | --- | --- | --- | --- | --- | --- |
|  | **Response Options** | | | | |  |  |
| **Prompt** | 1 | 2 | 3 | 4 | 5 | **Example** | |
| For the following questions, we will ask you how high or low the chances are the something will happen. | Very low | Low | Moderate | High | Very high | How high or low are the chances of you getting health problems from arsenic if it is in your well water? | |
| For these next questions, we will ask you how much you agree or disagree with a statement that we read. | Strongly disagree | Slightly disagree | Neither agree nor disagree | Slightly agree | Strongly agree | I have been drinking the water for a long time with no health problems, so I am not concerned about arsenic in my well water. | |
| For these questions, we will ask you about other people in your community (whatever you feel is your community). | None | A few of them | Half of them | Most of them | All | How many people in your community with arsenic in their wells drink this water without filtration? | |
| How I'm going to ask you some question about how much you approve or disapprove or something. | All disapprove | Some disapprove | Neither approve nor disapprove | Some approve | All approve | How much would people who are important to you approve or disapprove of you using water containing high arsenic for drinking? | |
| For these questions, we will ask you about how sure you are to do certain things. | 0% sure | 25% sure | 50% sure | 75% sure | 100% sure | How sure are you that you can get drinking water with a safe level of arsenic? | |
| Now I'm going to ask you some question about how committed you feel to certain things. | 0% | 25% | 50% | 75% | 100% | How committed do you feel to drinking water only from your arsenic filter faucet? | |
| For analysis, behavioral determinants were rescaled from original coding to a 0-1 scale. | | | | | | | |

| **Supplementary Table 2.** Differences in behavioral determinants at baseline by study arm | | | | | | | | | | |
| --- | --- | --- | --- | --- | --- | --- | --- | --- | --- | --- |
| **Statement** | **Behavioral determinant** | **mHealth & filter arm (N= 51)** | | | **Intensive arm (N = 33)** | | | **Difference** | | |
|  |  | Median | Mean | SD | Median | Mean | SD | Median | Mean | p-value |
| *How high or low are the chances of you getting health problems from arsenic if it is in your well water?* | Perceived vulnerability | 0.50 | 0.61 | 0.31 | 0.63 | 0.60 | 0.30 | 0.13 | -0.01 | 0.563 |
| *How high or low are the chances of someone in your household getting health problems from arsenic if it is in your well water?* | Perceived vulnerability | 0.50 | 0.63 | 0.30 | 0.63 | 0.59 | 0.31 | 0.13 | -0.05 | 0.286 |
| *How high or low are the chances of you getting health problems from arsenic if you drink water from an arsenic filter?* | Perceived vulnerability | 0.25 | 0.20 | 0.23 | 0.00 | 0.18 | 0.23 | -0.25 | -0.02 | 0.950 |
| *I have been drinking the water for a long time with no health problems, so I am not concerned about arsenic in my well water.* | Perceived vulnerability | 0.25 | 0.38 | 0.37 | 0.25 | 0.38 | 0.37 | 0.00 | 0.00 | 0.570 |
| *Because no one in my house has developed health problems from arsenic, I am not concerned about arsenic in my well water.* | Perceived vulnerability | 0.25 | 0.33 | 0.37 | 0.25 | 0.34 | 0.36 | 0.00 | 0.01 | 0.545 |
| *I can afford to fix my arsenic filter if it breaks.* | Perceived cost | 0.75 | 0.58 | 0.36 | 0.75 | 0.62 | 0.35 | 0.00 | 0.04 | 0.918 |
| *Of all the things I have to worry about, the arsenic filter is not at the top of my list.* | Competing Priorities | 0.38 | 0.46 | 0.36 | 0.25 | 0.37 | 0.37 | -0.13 | -0.08 | 0.604 |
| *If given the choice, I would prefer bottled water over water from an arsenic filter.* | User preferences | 0.50 | 0.52 | 0.36 | 0.50 | 0.41 | 0.37 | 0.00 | -0.11 | 0.295 |
| *The tribal water system in my area has a safe level of arsenic.* | Perceived safety of tribal water system | 0.50 | 0.33 | 0.27 | 0.50 | 0.41 | 0.32 | 0.00 | 0.08 | 0.300 |
| *How many people in your community have arsenic in their wells?* | Perceived extent of contamination in community | 0.63 | 0.61 | 0.27 | 0.75 | 0.58 | 0.27 | 0.13 | -0.03 | 0.458 |
| *How many people in your community with arsenic in their wells drink this water without filtration?* | Descriptive norm | 0.75 | 0.66 | 0.27 | 0.75 | 0.74 | 0.25 | 0.00 | 0.08 | 0.223 |
| *How many people in your community with arsenic in their wells cook with this water without filtration?* | Descriptive norm | 0.75 | 0.70 | 0.25 | 0.75 | 0.80 | 0.20 | 0.00 | 0.10 | 0.071 |
| **Cont. Supplementary Table 2.** Differences in behavioral determinants at baseline by study arm | | | | | | | | | | |
|  |  | **mHealth & filter arm (N= 51)** | | | **Intensive arm (N = 33)** | | | **Difference** | | |
| **Statement** | **Behavioral determinant** | Median | Mean | SD | Median | Mean | SD | Median | Mean | p-value |
| *How many people in your community with arsenic in their wells use bottled water for drinking?* | Descriptive norm | 0.25 | 0.31 | 0.18 | 0.25 | 0.30 | 0.27 | 0.00 | 0.00 | 0.644 |
| *How many people in your community with arsenic in their wells use an arsenic filter for their drinking water?* | Descriptive norm | 0.25 | 0.15 | 0.16 | 0.25 | 0.20 | 0.25 | 0.00 | 0.05 | 0.235 |
| *How many people in your community with arsenic in their wells use an arsenic filter for their cooking water?* | Descriptive norm | 0.25 | 0.17 | 0.22 | 0.25 | 0.23 | 0.29 | 0.00 | 0.06 | 0.182 |
| *How much would people who are important to you approve or disapprove of you using water containing high arsenic for drinking?* | Injunctive norm | 0.00 | 0.20 | 0.27 | 0.25 | 0.24 | 0.24 | 0.25 | 0.05 | 0.231 |
| *How much would people who are important to you approve or disapprove of you using water containing high arsenic for cooking?* | Injunctive norm | 0.13 | 0.21 | 0.27 | 0.25 | 0.26 | 0.24 | 0.13 | 0.05 | 0.240 |
| *How sure are you that you can get drinking water with a safe level of arsenic?* | Self-efficacy | 0.50 | 0.45 | 0.32 | 0.75 | 0.60 | 0.33 | 0.25 | 0.15 | 0.098 |
| *How sure are you that you can get water for cooking with a safe level of arsenic?* | Self-efficacy | 0.25 | 0.44 | 0.34 | 0.75 | 0.60 | 0.35 | 0.50 | 0.15 | 0.094 |
| *How sure are you that you could find local resources to learn about arsenic in water?* | Self-efficacy | **0.25** | **0.34** | **0.34** | **0.50** | **0.52** | **0.34** | **0.25** | **0.17** | **0.043** |
| *How sure are you that local resources would help you resolve an arsenic-related problem with your private well?* | Self-efficacy | 0.25 | 0.33 | 0.36 | 0.38 | 0.42 | 0.35 | 0.13 | 0.10 | 0.341 |
| *How sure are you that you can use your arsenic filter faucet every time you need water for drinking in your home?* | Self-efficacy | 1.00 | 0.85 | 0.23 | 1.00 | 0.90 | 0.18 | 0.00 | 0.05 | 0.184 |
| *How sure are you that you can use your arsenic filter faucet every time you need water for cooking in your home?* | Self-efficacy | 1.00 | 0.85 | 0.23 | 1.00 | 0.89 | 0.19 | 0.00 | 0.04 | 0.308 |
| *How sure are you that you will be able to buy a new arsenic filter cartridge when needed?* | Self-efficacy | 0.88 | 0.75 | 0.31 | 1.00 | 0.75 | 0.31 | 0.13 | 0.00 | 0.737 |
| **Cont. Supplementary Table 2.** Differences in behavioral determinants at baseline by study arm | | | | | | | | | | |
|  |  | **mHealth & filter arm (N= 51)** | | | **Intensive arm (N = 33)** | | | **Difference** | | |
| **Statement** | **Behavioral determinant** | Median | Mean | SD | Median | Mean | SD | Median | Mean | p-value |
| *How sure are you that you yourself can change your arsenic filter cartridge when needed?* | Self-efficacy | 0.75 | 0.67 | 0.36 | 1.00 | 0.73 | 0.38 | 0.25 | 0.07 | 0.287 |
| *How sure are you that you will be able to use your arsenic filter consistently over the next year?* | Self-efficacy | 1.00 | 0.92 | 0.18 | 1.00 | 0.94 | 0.17 | 0.00 | 0.02 | 0.295 |
| *How sure are you that you will be able to use your filter consistently over the next five years?* | Self-efficacy | 1.00 | 0.84 | 0.22 | 1.00 | 0.85 | 0.22 | 0.00 | 0.01 | 0.666 |
| *How committed do you feel to drinking water only from your arsenic filter faucet?* | Commitment strength | 0.75 | 0.75 | 0.30 | 1.00 | 0.86 | 0.20 | 0.25 | 0.10 | 0.119 |
| *How committed do you feel to cooking with water only from your arsenic filter faucet?* | Commitment strength | 1.00 | 0.82 | 0.25 | 1.00 | 0.83 | 0.23 | 0.00 | 0.01 | 0.789 |
| *Overall arsenic knowledge* | Knowledge | 0.71 | 0.63 | 0.29 | 0.71 | 0.64 | 0.32 | 0.00 | 0.00 | 0.878 |
| *Name two health conditions that can happen from arsenic exposure?* | Knowledge | 0.50 | 0.55 | 0.44 | 0.50 | 0.45 | 0.46 | 0.00 | -0.10 | 0.470 |
| *Name two tasks where it is OK to use water with high arsenic?* | Knowledge | 0.50 | 0.57 | 0.45 | 1.00 | 0.62 | 0.45 | 0.50 | 0.05 | 0.837 |
| *Name two tasks where it is NOT OK to use water with high arsenic?* | Knowledge | 1.00 | 0.73 | 0.38 | 1.00 | 0.73 | 0.40 | 0.00 | 0.00 | 0.797 |
| *How could you remove arsenic from drinking water?* | Knowledge | 1.00 | 0.78 | 0.42 | 1.00 | 0.85 | 0.36 | 0.00 | 0.06 | 0.395 |
| P-values calculated with regression with generalized estimating equations to account for household level clustering. P-value < 0.05 indicated in bold. Change = intensive arm - mHealth filter only arm. | | | | | | | | | | |

| **Supplementary Table 3.** Baseline, follow-up, and change over study period for behavioral determinants by study arm | | | | | | | | | | | | | |
| --- | --- | --- | --- | --- | --- | --- | --- | --- | --- | --- | --- | --- | --- |
| **Statement** | **Behavioral determinant** | **mHealth & filter arm** | | | | | | **Intensive arm** | | | | | |
|  |  | **Baseline (N = 51)** | | **Follow-up (N = 47)** | | **Change** | | **Baseline (N = 33)** | | **Follow-up (N = 28)** | | **Change** | |
|  |  | Mean | SD | Mean | SD | Mean | p-value | Mean | SD | Mean | SD | Mean | p-value |
| How high or low are the chances of you getting health problems from arsenic if it is in your well water? | Perceived vulnerability | 0.61 | 0.31 | 0.71 | 0.28 | 0.10 | 0.066 | 0.60 | 0.30 | 0.60 | 0.37 | -0.01 | 0.433 |
| How high or low are the chances of someone in your household getting health problems from arsenic if it is in your well water? | Perceived vulnerability | 0.63 | 0.30 | 0.69 | 0.29 | 0.06 | 0.284 | 0.59 | 0.31 | 0.59 | 0.36 | 0.00 | 0.369 |
| How high or low are the chances of you getting health problems from arsenic if you drink water from an arsenic filter? | Perceived vulnerability | 0.20 | 0.23 | 0.21 | 0.25 | 0.01 | 0.761 | 0.18 | 0.23 | 0.32 | 0.36 | 0.14 | 0.150 |
| I have been drinking the water for a long time with no health problems, so I am not concerned about arsenic in my well water. | Perceived vulnerability | 0.38 | 0.37 | 0.30 | 0.37 | -0.09 | 0.053 | 0.38 | 0.37 | 0.26 | 0.35 | -0.12 | 0.181 |
| Because no one in my house has developed health problems from arsenic, I am not concerned about arsenic in my well water. | Perceived vulnerability | 0.33 | 0.37 | 0.31 | 0.35 | -0.02 | 0.551 | 0.34 | 0.36 | 0.26 | 0.36 | -0.08 | 0.198 |
| I can afford to fix my arsenic filter if it breaks. | Perceived cost | 0.58 | 0.36 | 0.66 | 0.38 | 0.08 | 0.089 | 0.62 | 0.35 | 0.65 | 0.40 | 0.03 | 0.377 |
| Of all the things I have to worry about, the arsenic filter is not at the top of my list. | Competing Priorities | 0.46 | 0.36 | 0.52 | 0.38 | 0.07 | 0.407 | **0.37** | **0.37** | **0.63** | **0.41** | **0.25** | **0.021** |
| If given the choice, I would prefer bottled water over water from an arsenic filter. | User preferences | 0.52 | 0.36 | 0.47 | 0.37 | -0.05 | 0.967 | 0.41 | 0.37 | 0.40 | 0.33 | -0.01 | 0.981 |
| The tribal water system in my area has a safe level of arsenic. | Perceived safety of tribal water system | **0.33** | **0.27** | **0.49** | **0.31** | **0.16** | **0.000** | 0.41 | 0.32 | 0.48 | 0.32 | 0.07 | 0.056 |
| **Cont. Supplementary Table 3.** Baseline, follow-up, and change over study period for behavioral determinants by study arm | | | | | | | | | | | | | |
|  |  | **mHealth & filter arm** | | | | | | **Intensive arm** | | | | | |
|  |  | **Baseline (N = 51)** | | **Follow-up (N = 47)** | | **Change** | | **Baseline (N = 33)** | | **Follow-up (N = 28)** | | **Change** | |
| **Statement** | **Behavioral determinant** | Mean | SD | Mean | SD | Mean | p-value | Mean | SD | Mean | SD | Mean | p-value |
| How many people in your community have arsenic in their wells? | Perceived extent of contamination in community | 0.61 | 0.27 | 0.61 | 0.28 | 0.00 | 0.338 | 0.58 | 0.27 | 0.64 | 0.34 | 0.07 | 0.529 |
| How many people in your community with arsenic in their wells drink this water without filtration? | Descriptive norm | 0.66 | 0.27 | 0.61 | 0.29 | -0.06 | 0.091 | **0.74** | **0.25** | **0.62** | **0.28** | **-0.13** | **0.016** |
| How many people in your community with arsenic in their wells cook with this water without filtration? | Descriptive norm | 0.70 | 0.25 | 0.66 | 0.27 | -0.04 | 0.282 | **0.80** | **0.20** | **0.61** | **0.30** | **-0.20** | **0.000** |
| How many people in your community with arsenic in their wells use bottled water for drinking? | Descriptive norm | **0.31** | **0.18** | **0.42** | **0.27** | **0.11** | **0.008** | 0.30 | 0.27 | 0.34 | 0.28 | 0.03 | 0.664 |
| How many people in your community with arsenic in their wells use an arsenic filter for their drinking water? | Descriptive norm | **0.15** | **0.16** | **0.26** | **0.24** | **0.11** | **0.015** | 0.20 | 0.25 | 0.37 | 0.32 | 0.16 | 0.077 |
| How many people in your community with arsenic in their wells use an arsenic filter for their cooking water? | Descriptive norm | **0.17** | **0.22** | **0.27** | **0.25** | **0.10** | **0.017** | 0.23 | 0.29 | 0.32 | 0.30 | 0.09 | 0.277 |
| How much would people who are important to you approve or disapprove of you using water containing high arsenic for drinking? | Injunctive norm | **0.20** | **0.27** | **0.05** | **0.15** | **-0.15** | **0.017** | 0.24 | 0.24 | 0.21 | 0.28 | -0.03 | 0.804 |
| How much would people who are important to you approve or disapprove of you using water containing high arsenic for cooking? | Injunctive norm | **0.21** | **0.27** | **0.05** | **0.15** | **-0.16** | **0.016** | 0.26 | 0.24 | 0.21 | 0.27 | -0.05 | 0.449 |
| How sure are you that you can get drinking water with a safe level of arsenic? | Self-efficacy | **0.45** | **0.32** | **0.64** | **0.36** | **0.18** | **0.008** | 0.60 | 0.33 | 0.62 | 0.40 | 0.01 | 0.904 |
| How sure are you that you can get water for cooking with a safe level of arsenic? | Self-efficacy | **0.44** | **0.34** | **0.66** | **0.33** | **0.22** | **0.003** | 0.60 | 0.35 | 0.67 | 0.36 | 0.08 | 0.588 |
| **Cont. Supplementary Table 3.** Baseline, follow-up, and change over study period for behavioral determinants by study arm | | | | | | | | | | | | | |
|  |  |  | **mHealth & filter arm** | | | | | **Intensive arm** | | | | | |
|  |  | **Baseline (N = 51)** | | **Follow-up (N = 47)** | | **Change** | | **Baseline (N = 33)** | | **Follow-up (N = 28)** | | **Change** | |
| **Statement** | **Behavioral determinant** | Mean | SD | Mean | SD | Mean | p-value | Mean | SD | Mean | SD | Mean | p-value |
| How sure are you that you could find local resources to learn about arsenic in water? | Self-efficacy | **0.34** | **0.34** | **0.53** | **0.35** | **0.19** | **0.000** | 0.52 | 0.34 | 0.55 | 0.39 | 0.03 | 0.785 |
| How sure are you that local resources would help you resolve an arsenic-related problem with your private well? | Self-efficacy | **0.33** | **0.36** | **0.51** | **0.37** | **0.18** | **0.003** | 0.42 | 0.35 | 0.46 | 0.35 | 0.04 | 0.951 |
| How sure are you that you can use your arsenic filter faucet every time you need water for drinking in your home? | Self-efficacy | 0.85 | 0.23 | 0.79 | 0.29 | -0.06 | 0.140 | **0.90** | **0.18** | **0.87** | **0.23** | **-0.04** | **0.011** |
| How sure are you that you can use your arsenic filter faucet every time you need water for cooking in your home? | Self-efficacy | 0.85 | 0.23 | 0.82 | 0.27 | -0.04 | 0.354 | **0.89** | **0.19** | **0.88** | **0.24** | **-0.02** | **0.025** |
| How sure are you that you will be able to buy a new arsenic filter cartridge when needed? | Self-efficacy | 0.75 | 0.31 | 0.67 | 0.34 | -0.08 | 0.236 | 0.75 | 0.31 | 0.70 | 0.29 | -0.05 | 0.247 |
| How sure are you that you yourself can change your arsenic filter cartridge when needed? | Self-efficacy | **0.67** | **0.36** | **0.54** | **0.40** | **-0.13** | **0.033** | 0.73 | 0.38 | 0.79 | 0.36 | 0.05 | 0.550 |
| How sure are you that you will be able to use your arsenic filter consistently over the next year? | Self-efficacy | 0.92 | 0.18 | 0.86 | 0.24 | -0.06 | 0.082 | **0.94** | **0.17** | **0.91** | **0.23** | **-0.03** | **0.020** |
| How sure are you that you will be able to use your filter consistently over the next five years? | Self-efficacy | **0.84** | **0.22** | **0.78** | **0.32** | **-0.06** | **0.030** | 0.85 | 0.22 | 0.83 | 0.29 | -0.02 | 0.114 |
| How committed do you feel to drinking water only from your arsenic filter faucet? | Commitment strength | 0.75 | 0.30 | 0.76 | 0.30 | 0.01 | 0.814 | **0.86** | **0.20** | **0.83** | **0.24** | **-0.03** | **0.039** |
| **Cont. Supplementary Table 3.** Baseline, follow-up, and change over study period for behavioral determinants by study arm | | | | | | | | | | | | |  |
|  |  | **mHealth & filter arm** | | | | | | **Intensive arm** | | | | | |
|  |  | **Baseline (N = 51)** | | **Follow-up (N = 47)** | | **Change** | | **Baseline (N = 33)** | | **Follow-up (N = 28)** | | **Change** | |
| **Statement** | **Behavioral determinant** | Mean | SD | Mean | SD | Mean | p-value | Mean | SD | Mean | SD | Mean | p-value |
| How committed do you feel to cooking with water only from your arsenic filter faucet? | Commitment strength | 0.82 | 0.25 | 0.78 | 0.30 | -0.04 | 0.422 | 0.83 | 0.23 | 0.87 | 0.25 | 0.03 | 0.412 |
| Overall arsenic knowledge | Knowledge | 0.63 | 0.29 | 0.70 | 0.26 | 0.07 | 0.252 | **0.64** | **0.32** | **0.56** | **0.32** | **-0.08** | **0.030** |
| Name two health conditions that can happen from arsenic exposure? | Knowledge | 0.55 | 0.44 | 0.59 | 0.41 | 0.04 | 0.713 | 0.45 | 0.46 | 0.46 | 0.40 | 0.01 | 0.395 |
| Name two tasks where it is OK to use water with high arsenic? | Knowledge | 0.57 | 0.45 | 0.65 | 0.44 | 0.08 | 0.589 | 0.62 | 0.45 | 0.54 | 0.48 | -0.08 | 0.093 |
| Name two tasks where it is NOT OK to use water with high arsenic? | Knowledge | 0.73 | 0.38 | 0.80 | 0.38 | 0.07 | 0.676 | **0.73** | **0.40** | **0.59** | **0.42** | **-0.13** | **0.029** |
| How could you remove arsenic from drinking water? | Knowledge | 0.78 | 0.42 | 0.84 | 0.37 | 0.06 | 0.097 | **0.85** | **0.36** | **0.78** | **0.42** | **-0.07** | **0.026** |
| P-values calculated with regression with generalized estimating equations to account for household level clustering. P-value < 0.05 indicated in bold. Change = intensive arm-mHealth filter only arm. | | | | | | | | | | | | | |

| **Supplementary Table 4.** Influence of demographic factors on follow-up use of arsenic-safe water | | | | | | | | | |
| --- | --- | --- | --- | --- | --- | --- | --- | --- | --- |
|  | **Exclusive use of arsenic-safe water** | | | | | | | | |
|  | **Cooking** | | | **Drinking** | | | **Cooking and drinking** | | |
| **Demographic factor** | OR | 95% CI | p-value | OR | 95% CI | p-value | OR | 95% CI | p-value |
| *Male* | 0.56 | 0.25, 1.24 | 0.154 | 0.71 | 0.37, 1.39 | 0.317 | 0.61 | 0.28, 1.31 | 0.206 |
| *Education* | 2.17 | 0.94, 5.00 | 0.069 | 1.54 | 0.72, 3.31 | 0.270 | 1.54 | 0.70, 3.40 | 0.282 |
| Age |  |  |  |  |  |  |  |  |  |
| *12-34* | 1 (Ref) | - | - | - | - | - | - | - | - |
| *35-64* | 2.07 | 0.58, 7.37 | 0.262 | 0.53 | 0.20, 1.37 | 0.187 | 0.82 | 0.28, 2.39 | 0.650 |
| *≥ 65* | 1.41 | 0.39, 5.07 | 0.603 | 0.85 | 0.29, 2.48 | 0.759 | 0.76 | 0.23, 2.51 | 0.720 |
| Odds ratios calculated with regression with generalized estimating equations to account for household level clustering. P-value < 0.05 indicated in bold. Education compares high school or less education to more than high school education. | | | | | | | | | |

| **Supplementary Table 5.** Influence of demographic factors on arsenic filter cartridge change | | | |
| --- | --- | --- | --- |
|  | **Arsenic filter cartridge change** | | |
| **Demographic factor** | OR | 95% CI | p-value |
| *Male* | 1.06 | 0.50, 2.22 | 0.887 |
| *Education* | 0.37 | 0.13, 1.03 | 0.056 |
| Age |  |  |  |
| *12-34* | 1 (Ref) | - | - |
| *35-64* | 0.40 | 0.10, 1.62 | 0.201 |
| *≥ 65* | 0.26 | 0.05, 1.51 | 0.135 |
| Odds ratios calculated with regression with generalized estimating equations to account for household level clustering. P-value < 0.05 indicated in bold. | | | |
